# A Novel Method to Analyze Social Transmission in Chronologically Sequenced Assemblages, Implemented on Cultural Inheritance of the Art of Cooking

**DOI:** 10.1371/journal.pone.0122092

**Published:** 2015-05-13

**Authors:** Sven Isaksson, Alexander Funcke, Ida Envall, Magnus Enquist, Patrik Lindenfors

**Affiliations:** 1 Centre for the Study of Cultural Evolution, Stockholm University, Stockholm, Sweden; 2 Archaeological Research Laboratory, Stockholm University, Stockholm, Sweden; 3 Philosophy, Politics and Economics, University of Pennsylvania, Philadelphia, Pennsylvania, United States of America; 4 Department of Zoology, Stockholm University, Stockholm, Sweden; Durham University, UNITED KINGDOM

## Abstract

Here we present an analytical technique for the measurement and evaluation of changes in chronologically sequenced assemblages. To illustrate the method, we studied the cultural evolution of European cooking as revealed in seven cook books dispersed over the past 800 years. We investigated if changes in the set of commonly used ingredients were mainly gradual or subject to fashion fluctuations. Applying our method to the data from the cook books revealed that overall, there is a clear continuity in cooking over the ages – cooking is knowledge that is passed down through generations, not something (re-)invented by each generation on its own. Looking at three main categories of ingredients separately (spices, animal products and vegetables), however, disclosed that all ingredients do not change according to the same pattern. While choice of animal products was very conservative, changing completely sequentially, changes in the choices of spices, but also of vegetables, were more unbounded. We hypothesize that this may be due a combination of fashion fluctuations and changes in availability due to contact with the Americas during our study time period. The presented method is also usable on other assemblage type data, and can thus be of utility for analyzing sequential archaeological data from the same area or other similarly organized material.

## Introduction

In this article we present an analytical technique for the measurement and evaluation of change in distributions of chronologically sequenced assemblages. This method can be applicable to e.g. well-dated archaeological finds. We opted for Euclidean distance as a measure of dissimilarity in this paper, although choice of distance measure can be adapted to hypothesis and data. By standardizing the Euclidean distance to the distance in time between sequential neighbors this technique allows investigation of the change in change itself. Though methods to investigate the pace of cultural evolution are not uncommon [1, 2, 3, 4], to the best of our knowledge ours is the first method that is useable on assemblages, where the data consists of frequencies and records of existence/non-existence of items. We illustrate the method by investigating the cultural evolution of cooking as revealed in seven cook books dispersed over the past 800 years.

Food is something that humans of every age have engaged in heartily, preferably on a daily basis. Eating is a biological necessity—within about twelve hours since the last meal humans typically begin to feel hunger, and within approximately twenty hours the human body begins to adjust physiologically and enter a state of starvation [5]. Thus, people need to eat from biological necessity, but food and eating also contains a strong cultural component. *Experiences* of tastes, either good or bad, are sensory impressions, but *preferences* of taste, again good or bad, are also something that is learnt, from others and through experience—the characteristic of tasting either good or bad is not an inherent quality of any victuals.

Consequently, the list of articles that, far and wide in time and space, have been considered as foods is truly massive [6, 7, 8]. In fact, humans display a remarkable capacity to be able to live on a vast range of very different diets and it is through this capacity of dietary diversity that humans, as the only mammal in our size-range, has managed to chisel out a living from almost every environment on the planet. Nevertheless, humans typically do not eat everything edible in their environment at any specific time or place, not even in the face of famine. This human aptitude for cultural diversity in diet makes food and eating suitable topics for the study of cultural evolution.

In the present paper we report the results from a comparative study on ingredients used in a selection of northwestern European cookery books, spanning the late medieval period into the late modern era. There exist several earlier studies on changes in food culture, from different and sometimes opposing perspectives [9, 10], but most of the more recent food culture studies are focused on particular aspects, such as gender [11, 12], emotions [13] or ethnic identity [14], though there are also examples of studies concerned with general patterns of change [15, 16]. When evolution is included in the study, the focus is usually on biology [17] rather than culture. We do not deny the relevance of these more particular approaches, but our research interests here concern patterns of cultural change on a more general timescale.

The value of recipe collections for the study of short-term changes in modern cookery books has already been shown (e.g. [18]). Of importance for our study is the reasonably long history of cookery books as well as their suitability for systematical comparative approaches. Thus far we have developed a method for textual analysis whereby we systematically record recipes in cookery books as algorithms, to facilitate application of statistical analyses [15]. In another study of the material, we identified increases in the complexity of cooking over the last 800 years in the number of steps, techniques, ingredients, and semi-manufactured ingredients. In the present study we instead focus on the question if change in cooking is gradual or saltational, and if we can identify any clear breaks in the material between different age-bound traditions.

Food habits have been characterized as something very conservative, perceived as changing only slowly over the centuries and as something to be studied in the perspective of the *longue durée* [19]. This is an emphasis on transmission of customs and knowledge from one generation to the next. However, in food and cookery changes occur also through lending from other cooking-traditions, distortion (i.e. copying or transmission errors), fashions (i.e. fluctuating changes in the popularity of recipes and ingredients unrelated to the recipes and ingredients themselves) or innovation (i.e. individual learning). If lending takes place from groups with prestige this may result in a quick, widespread changes (i.e. food fashions), whereas copying from peers (e.g. neighbors of a similar station in life) may be slower and more local. Distortion is the slow and subtle change that can take place in dishes over time, in which ingredients may increase or decrease in amount, become excluded or exchanged, or new ones included [15].

We made the following predictions: If there is a strong emphasis on social learning from earlier generations, similarity should be greatest between sequential neighbors in a chronological sequence. Food categories more exposed to quick changes due to learning from other food-traditions, to fashions or to innovations, would be identified by their *not* having the greatest similarities with sequential neighbors. We predict that basic food stuffs, such as animal products and vegetables, should change more gradually as compared to spices that should be more subject to fashion fluctuations. We thus posed the following two questions: (1) Is the evolution of cooking in general gradual or saltational, and (2) do ingredients of different categories (e.g. animal products and vegetables vs. spices) change differently over time—can we trace different modes of cultural evolution in cooking?

## A Brief Cultural History of Cooking

To sum up the cultural history of cooking, even when limited to northwestern Europe, is challenging—in fact, it is a subject that fills books (e.g. [8, 20, 21]). Nonetheless, some generalities can be observed. The cultural history of cooking is to some extent characterized by the availability of foodstuffs, which in turn is dependent on both regional differences (e.g. [22]) as well as on preservation techniques and transport. As we have chosen to study one region (i.e. Northwestern Europe), regional differences may be presumed to have a minor effect. However, social differences cannot be discounted similarly. For example, for most of European history grains have accounted for more than 50% of the caloric intake, for the lower classes even reaching 70–75% at times. Cookery books from various social strata may therefore show a dissimilarity that is dependent on social difference rather than on cultural evolutionary processes.

The food culture of the Medieval Period was profoundly shaped by various ecclesiastic decrees, but also includes influences from Arabic food culture, mainly through Sicily and Spain rather than through the Crusades. Most food was cooked over an open fire in the fireplace, using fitting cooking techniques, and much everyday food in the ordinary household was in the form of a one-pot stew. Preservation techniques available, in various degrees, were drying, smoking, pickling, salting, fermentation and smoking.

The Renaissance saw the introduction of new foodstuffs brought in from the New World as well as the rediscovery of many old foodstuffs and of new table-manners, e.g. the introduction of knife-&-fork. Up until the French Revolution such news spread mainly through royal courts and aristocracy, trailing political fluctuations of influence and contact. However, for most people, much of the European food history was characterized by the threat of famine. The public authorities were often seen as guarantors of food by their subjects and when this failed revolts and food riots followed. As a result there is a period of great food conflicts, accompanied by equally great martial conflicts (e.g. The Thirty Years’ War), that runs from the early 17^th^ century to the first decades of the 19^th^, though varying greatly between regions.

After the social convulsions of the French Revolution the prominent cooks found their new place in a commencing catering trade, operating restaurants and hotels sustained by bourgeois people finding the means for an extravagant lifestyle in the produce of the ongoing industrialization. The ways of presenting food changed during the 19^th^ century, from the collective servings of many dishes to the presentation of dishes one by one. Around the mid-19^th^ century there was a major change in cooking as the iron rage or stove quickly replaced the earlier open fireplace. Around the same time canning of food as a means of preservation had its breakthrough and sugar stopped being exclusively a luxury food. The domination of grains in the European diet began to decline from the middle decades of the 19^th^ century, while other foods, especially meat, increased.

After 1900 came a simplification of food decoration (*Cuisine classique*) and innovative changes in the organization and division of labor in the restaurant kitchens. An idea of a “democratic” consumption of food, governed by the-freedom-of-choice, began to spread and social difference came to be expressed more and more through quality rather than through kind and quantity. From the early 20^th^ century refrigerators and freezers got more and more common in food storage. By the end of the 1950s a cuisine emerged maintaining nutritional benchmarks, characterized by a neo-simple tenseness, and more careful preparation together with artistic displays (*Nouvelle cuisine*). The late modern period is characterized by an increased internationalization regarding foodstuffs, recipes and cooking techniques, as well as an increase in semi manufactured products, much of it for microwave ovens. Such new household technologies have been comprehended as simplifications of cooking but have instead increased the variation in what we eat rather than decreasing the time spent cooking [23]. A novelty in preservation from this late period, getting more and more common, is the irradiation of foodstuffs. The advances in food transport and preservation has now all but erased the connections in eating between territory, season and food. Along these lines European food culture underwent degrees of normalization, food customs now being more socially determined rather than regionally. However, more traditional menus were still often preferred at banquets and solemn feasts and festivities. In the restaurant world there was a trend to pick up elements of *cuisine classique* style of cooking again, while keeping much of the lighter presentations and new techniques of the *nouvelle cuisine*.

## Materials and Methods

### Selection of cookery books

Northwestern Europe was selected as the area of investigation because of the comparatively rich source material in the region and the relatively easy access to this source material. We limited our choice of older cookery books to books available in English or Swedish, as we did not possess knowledge of other ancient or contemporary languages. As with all written sources, even cookery books are subject to source critical problems that we have had to deal with. One such problem is that several of the manuscripts surviving from the early periods of our study are actually different transcripts of the same original. To resolve this problem we have relied on the skillful work of the scholars who have analyzed and published these texts. Another basic source problem is the situation of production, which for the cookery books in our study certainly have changed over time. The early recipes consist mostly of short notes for memory of professional cooks in feudal aristocratic and royal settings, whereas the more recent ones are intended for a broader public. This may introduce a bias of more advanced cooking represented in the early cookery books. Also, the upper-class manuscripts contain recipes for both complex costly dishes and simpler everyday ones. Analogously, there are recipes for both everyday cooking as well as festivity food to be found in the late more common cookery books. Lastly, whether the message is open or hidden, even cookery books may have been written with an agenda, with the intention to change ideas and behavior of the readers. Such an agenda may well have an ideological stimulus reaching far beyond the immediate culinary sphere.

A cookery book is thus not merely a rendering of the culinary practices of the time and place of its creation. In fact, the occurrence of a recipe in that cookery book is no guarantee for it to have ever been prepared. Many cookery books may certainly portray what people have wished they could cook and eat [11]. However, the cookery books that we have included in our study we know, or have good reasons to believe, have been in actual practical use.

For this study we have chosen to focus on northwestern Europe in general and Scandinavia in particular, for the sake of regional consistency. The oldest cookery book included is also one of the oldest surviving post-Roman cookery manuscript, most likely composed in the first half of the 13^th^ century: *Libellus de arte coquinaria* (The Little Book of Culinary Arts). It is preserved as four related manuscripts written in Old Danish, Icelandic and Low German, all probably based on a slightly earlier, possibly French manuscript [24].

Following this and preceding the printed cookery books are some 150 other surviving manuscripts in various languages. However, many of these can also be grouped into families, being based on a common ancestral compilation of recipes, itself often lost, making the number of separable compilations much smaller [25]. From this period we have chosen to include the *Forme of Cury* (Forms of Cooking, *cury* being from French), a collection of some 250 recipes of the 14^th^ century, whose author is given as "the chief Master Cooks of King Richard II", an example of recipe collections of professional cooks in an aristocratic setting.

After the first printed cookery book, the Italian *De honesta voluptate* from 1475 [26], the number of available documents increases. During the Renaissance Italy was in this sense the leading country, while the French cookery book production flourished in the 17^th^ century and have had a strong position since. The numerous German cookery books from the 16^th^ and 17^th^ century had a strong influence on Swedish cooking. From Sweden there are handwritten compilations of recipes preserved from the 17^th^ century, and the earliest printed one is from 1642. As an example from this period, we have selected the anonymous *Een Lijten Kockebook* (A Small Cookery Book) from 1650 [27], which probably is compiled from German originals. *Een Lijten Kockebook* describes a comparatively homely cooking.

From the 18^th^ century there are several Swedish cookery books, many of which may be classified as “housekeeping books” containing recipes for much more than the cooking of foods. Of these we have chosen one written by Gustaf Abraham Piper and presented to his young wife Märta Sture as a gift in 1739, contains 368 recipes of all sorts of food, drinks, etc. [28]. This and the previous one are both set within the period of great food conflicts (early 17^th^ to early 19^th^ century; Montanari, 1998:108).

There are numerous cookery books from the 19^th^ and 20^th^ centuries, though during the 2^nd^ half of the 20^th^ century the role of the cookery book has to some extent been taken over by food magazines and internet sites. From late 19^th^ century we have chosen C. E. Hagdahls book *Kok-konsten som vetenskap och konst* (The Culinary Arts as Science and Art) [29], looked upon as the book that introduced modern cooking to Sweden. It contains 3000 recipes, and is a momentous summary of European culinary arts of the time. Moreover, it introduces a new thinking as to the physiology of nutrition. This is a cookery book with an agenda, definitely written with an intention to change people’s behavior in relation to food.

From the 20th century we have selected two books. *Kajsas Kokbok* (Kajsa’s Cookery Book) was first published in the 1930s and this is the 24^th^ edition, from 1976 [30]. It originally had the subtitle “a cookery book for the country household”. The late modern era is represented by another Swedish classic, the *Vår Kokbok* (Our Cookery Book), published by the Swedish Coop, or Consumer Cooperative Society, here in its 22^nd^ edition from 1999 [31]. Aimed for a broad public this cookery book was written in a new simpler way, changing the standard of writing recipes, and represents a late modern fairly urbanized cooking.

### Methods

Our approach is quantitative and statistical, aiming at empirical generalizations based on chronological series of data (see also [28]). Earlier methods used to investigate the pace of cultural evolution have focused on the cultural evolution of individual traits [1, 2, 3], not assemblages as is the case here, sometimes even assuming phylogenetic relatedness of cultural traits [4]. To the best of our knowledge ours is the first method that useable for comparing frequency counts in sequentially ordered assemblages. Thus, our proposed method is not comparable to earlier method-contributions as these do not incorporate options for studying changes in assemblages (e.g. [1, 2, 3, 4]).

To illustrate the method, we aimed at coding ten recipes from each of the categories”poultry recipes”,”fish recipes” and”red meat recipes”, arbitrarily selected from each cookery book, though all cookery books did not contain that many recipes in each category (Table 1).

**Table 1:** The cookery books, their date (sometimes approximate), and the number of recipes included from each in this study.

Year	Book	Number of Recipes
1200	*Libellus de arte coquinaria*	17
1390	*Forme of cury*	29
1650	*Een Lijten Kockebok*	28
1739	*Märta Stures hushållsbok*	28
1879	*Kok-konsten som vetenskap och konst*	30
1976	*Kajsas kokbok*	30
1999	*Vår kokbok*	30

For each recipe the number of methods/techniques, the number of steps in the preparation process, the number of ingredients, the number of semi-manufactured ingredients (defined as prepared ingredients, containing one or more raw products), the number of compound semi-manufactured ingredients (defined as semi-manufactured ingredients containing no less than two raw products), and the number of self-made semi-manufactured ingredients were counted. Moreover, the number of separate partial processes was counted [15].

In total 302 different ingredients were used in these 192 recipes, but many of them only occur occasionally. A first selection of ingredients was extracted setting a threshold of occurrence in at least 10 percent of the recipes of any given cookery book. This resulted in 76 different ingredients, many still quite sporadic in occurrence. This selection was used for the investigation of changes in the three categories animal products, vegetables and spices. An even stricter threshold at 35 percent of occurrence in the recipes of any given cookery book was also used and resulted in 22 different ingredients. This latter selection was used to investigate general trends.

To do the analysis we calculated the relative frequency of the ingredients in each cookery book, that is if for each ingredient *i* and cookery book *b*, we have a relative frequency *f*_*b*,*i*_ = *n*_*b*,*i*_/*N*_*b*_, where *n*_*b*,*i*_ is the number of recipes in cookery book *b* containing ingredient *i*, and *N*_*b*_ is the total number of recipes in book *b*.

Next we considered each cookery book as a point in the Euclidean space spanned by letting each ingredient frequency be a dimension, hence cookery book *b* is the point $\vec{b}=(f_{b,1},\ldots ,f_{b,m})$, where *m* is the number of ingredients considered. Our interest is in how cookery books change over time, hence the difference between books is what matters, to measure this we employ the so-called Euclidean distance; $\delta (\vec{a},\vec{b})=\sqrt{{\left(f_{a,1}–f_{b,1}\right)}^{2}+\ldots +{\left(f_{a,m}–f_{b,m}\right)}^{2}}\leq \sqrt{m}$. For convenience and for intuitive comparability, the distance is normalized, $\hat{\delta }(\vec{a},\vec{b})=\delta \left(\vec{a},\vec{b}\right)/\sqrt{m}$, where *a* is the second cook book considered. This gives us a sense of how different cookery books are. The books are, however, far from evenly distributed in time, and hence it is not straightforward to analyze pace of change.

To account for the varying time distribution a final metric was constructed. We did not simply divide by the time elapsed, as even two cookery books released the same day ought to vary quite a bit. To estimate a natural background variation we instead calculated the weighted minimal distance (with the least amount of difference between assemblages) between any two books $d_{min}={min}_{\vec{a},\vec{b}}\hat{\delta }(\vec{a},\vec{b})(1-\gamma )$, over all $\vec{a},\vec{b}$, and where 0 ≤ *γ* ≤ 1 is an estimation of how much of the difference between cookery books *a* and *b* is due to the time factor. The final metric is therefore, $d(a,b)=[\hat{\delta }(a,b)–d_{min}(1-\gamma )]\frac{t_{max}–t_{min}}{\left|t_{b}-t_{a}\right|}$, where the various *t* are publishing dates for the books. At λ = 1.0 all the difference is estimated to be due to the time difference and at λ = 0.1 only 10% of the minimal distance between any two cookery books is estimated to be due to the time difference. Lastly, the metric was again normalized, this time in respect to the total time-span considered, that is *t*_*max*_−*t*_*min*_.

## Results

**Fig 1:**
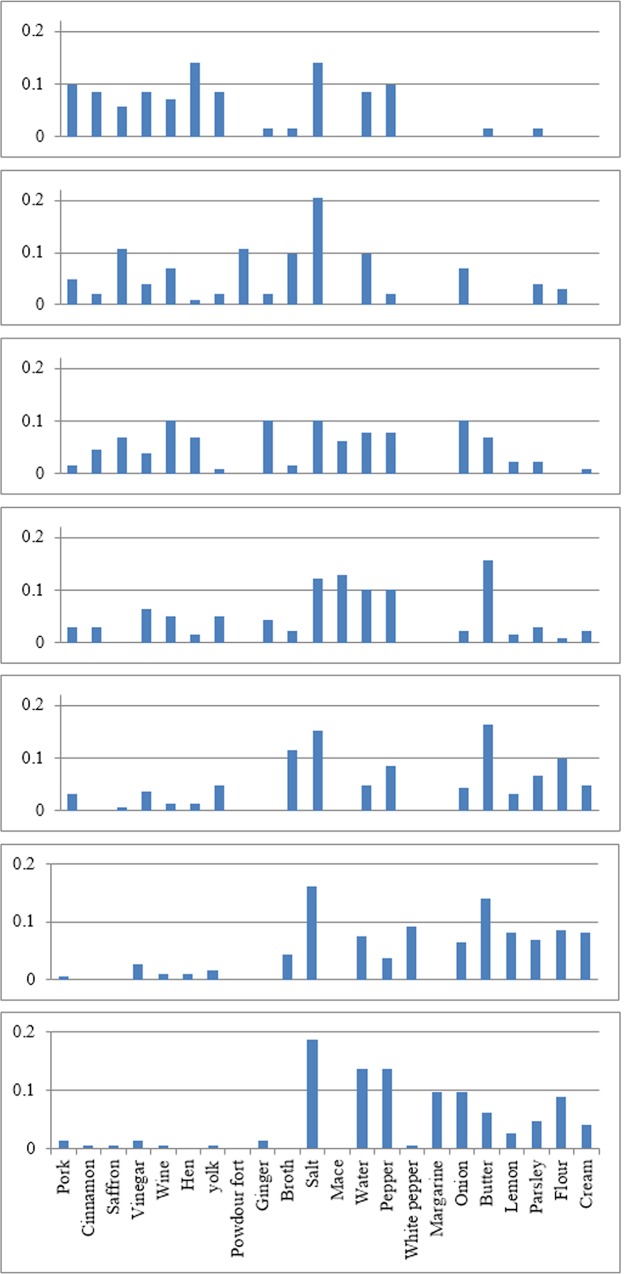
The distribution of ingredients that are present in at least 35% of the recipes in the different cookery books (here indicated by their date, Table 1), sorted from left to right in order of how common they are in different cookery books—ingredients more common in earlier books are on the left, ingredients more common later are on the right.

The 22 ingredients present in 35% of the recipes of any given cookery book were, in order of increasing commonness later in time: Pork, Cinnamon, Saffron, Vinegar, Wine, Hen, Yolk, Powdour fort, Ginger, Broth, Salt, Mace, Water, Pepper, White pepper, Margarine, Onion, Butter, Lemon, Parsley, Flour and Cream (Fig 1).

We compared the distributions (Fig 1) by calculating the relative Euclidean distance, which resulted in the matrices presented in Table 2 (topmost table). In general, we found the dissimilarity to be smallest between sequential neighbors in six out of seven cases; the exception being that the 1200 cook book is more similar to the 1650 cook book than the one from 1390. The greatest change per time unit in the pre-modern era occurs between 1650 and 1739, however dwarfed by the rate of change between 1976 and 1999 (Table 3). This latter high rate of change, however, is probably due to a high noise to signal ratio since these two cookery books are so closely located in time.

**Table 2:** Chronologically ordered matrices of normalized relative Euclidean distances between cookery books based on the distributions of ingredients (Fig 1).

All ingredients	1200	1390	1650	1739	1879	1976	1999
1200	0.00	5.59	**4.75**	5.61	6.40	6.89	6.40
1390	5.59	0.00	**5.18**	6.42	5.78	6.03	5.80
1650	4.75	5.18	0.00	**4.09**	5.71	5.69	5.44
1739	5.61	6.42	**4.09**	0.00	4.54	5.07	5.39
1879	6.40	5.78	5.71	4.54	0.00	**3.26**	4.84
1976	6.89	6.03	5.69	5.07	**3.26**	0.00	4.54
1999	6.40	5.80	5.44	5.39	4.84	**4.54**	0.00
Spices	1200	1390	1650	1739	1879	1976	1999
1200	0.00	9.53	**6.85**	9.35	9.47	11.93	7.91
1390	9.53	0.00	**8.40**	11.84	12.23	12.09	11.88
1650	6.85	8.40	0.00	**6.70**	10.28	11.44	9.05
1739	9.35	11.84	**6.70**	0.00	9.75	11.70	8.92
1879	9.47	12.23	10.28	9.75	0.00	9.99	**5.68**
1976	11.93	12.09	11.44	11.70	9.99	0.00	**9.70**
1999	7.91	11.88	9.05	8.92	**5.68**	9.70	0.00
Animal products	1200	1390	1650	1739	1879	1976	1999
1200	0.00	**8.30**	8.47	11.06	11.26	12.47	10.59
1390	**8.30**	0.00	9.99	10.59	10.82	11.50	8.40
1650	8.47	9.99	0.00	**7.41**	7.60	8.15	8.44
1739	11.06	10.59	7.41	0.00	**2.78**	5.11	7.58
1879	11.26	10.82	7.60	**2.78**	0.00	3.77	6.94
1976	12.47	11.50	8.15	5.11	**3.77**	0.00	6.42
1999	10.59	8.40	8.44	7.58	6.94	**6.42**	0.00
Vegetable products	1200	1390	1650	1739	1879	1976	1999
1200	0.00	**16.47**	17.63	18.29	17.67	17.80	17.00
1390	16.47	0.00	**7.91**	8.42	8.77	9.80	8.00
1650	17.63	**7.91**	0.00	8.65	9.02	9.10	7.77
1739	18.29	**8.42**	8.65	0.00	10.04	10.90	10.22
1879	17.67	8.77	9.02	10.04	0.00	**5.08**	3.97
1976	17.80	9.80	9.10	10.90	5.08	0.00	**4.61**
1999	17.00	8.00	7.77	10.22	**3.97**	4.61	0.00

The tables show distances for All ingredients (n = 22), Spices (n = 20), Animal products (n = 21) and Vegetables (n = 21) present in at least 35% (All ingredients) or 10% (Spices, Animal products & Vegetables) of recipes of any cookery book. Distances are expressed as a percentage of the maximum distance, making the matrices comparable. The distance from each cook book to the one it is most similar to is marked per row with **bold text**, and the distance from each cook book it is least similar to is marked with underlined text. The shorter the distance (the lower the value), the more similar the cook books with regards to the ingredients specified in the top left corner of each table.

**Table 3:** The rate of change (i.e. dissimilarity per time unit, cf. formulas above) in the distribution of ingredients present in 35% of the recipes of any of the included cookery books (All ingredients) or 10% of the recipes (Spices, Animal products and Vegetable products) at different estimates of how much of the difference between cookery books that is due to the time factor (λ).

λ	1200–1390	1390–1650	1650–1739	1739–1879	1879–1976	1976–1999
	*All ingredients*					
1.0	0.235	0.167	0.372	0.229	0.274	1.65
0.5	0.165	0.116	0.222	0.174	0.137	1.07
0.1	0.108	0.075	0.103	0.098	0.027	0.613
	*Spices*					
1.0	0.401	0.258	0.601	0.556	0.823	3.37
0.5	0.281	0.171	0.346	0.394	0.589	2.38
0.1	0.186	0.101	0.142	0.264	0.402	1.59
	*Animal products*					
1.0	0.336	0.314	0.650	0.162	0.288	2.28
0.5	0.277	0.270	0.523	0.081	0.171	1.78
0.1	0.229	0.235	0.421	0.016	0.078	1.39
	*Vegetable products*					
1.0	0.637	0.242	0.789	0.587	0.428	1.64
0.5	0.552	0.180	0.607	0.471	0.261	0.939
0.1	0.484	0.130	0.461	0.379	0.127	0.376

At λ = 1.0 all the difference is estimated to be due to the time difference and at λ = 0.1 only 10% of the minimal distance (with the least amount of difference between assemblages) between any two cookery books is estimated to be due to the time difference.

We divided the dataset based on the threshold of presence in at least 10% of recipes into three different categories; spices (n = 20), animal products (n = 21) and vegetable products (n = 21) (S1 Appendix and Table 1). These were analyzed separately through Euclidean distances in chronological matrices for each category (Table 2, bottom three tables). For animal products similarity was greatest between sequential neighbors in seven out of seven cases, while we found less gradual change in the usage of vegetables (five of seven) and spices (four of seven). Again, the greatest change per time unit in the pre-modern era occurs between 1650 and 1739 in all three categories, also here dwarfed by the rate of change between 1976 and 1999 (Table 3).

## Discussion

Our method of comparing Euclidean distances of the chronologically sequenced assemblages (the seven cookery books) indicated a connectedness in time in the use of ingredients at least from the 14^th^ century up till late 20^th^ century. In six out of seven columns in Table 2, the most similar distribution is found with a cookery book directly adjacent in time. This indicates a general pattern of learning from earlier generations regarding the use of these ingredients. The 13^th^ century cookery book, however, deviated slightly from this norm. This may indeed illustrate a small brake in social learning, or it may be due to the fact that this manuscript is the most fragmented in the study. A somewhat larger change can also be seen between the 1739 and 1879 cookery books (Table 2), as the cookery books from 1739 and earlier are more similar to earlier cookery books while the book from 1879 and later are more similar to later cookery books, potentially marking a shift from a pre-modern, pre-industrial era to the modern industrial era. Overall, however, our data indicates is a continuity in cooking over the ages, a result indicating that cooking is generally something handed down from generation to generation rather than subject to large fashion fluctuations. This method is also applicable on other assemblage-type data, e.g. finds from archaeological digs.

The chronological matrices for the three main categories of ingredients (spices, animal products and vegetables) indicated the presence of interesting special cases. In the table on spices, adjacent cookery books were most similar in only four out of seven cases (Table 2, second table from top), indicating that choice of spices may be more subject to fashion fluctuations or innovations than other ingredients. Compare this with the table on different animal (terrestrial and aquatic) products (Table 2, third table from top), where the most similar cookery books are adjacent in time in all seven out of the seven columns, indicating learning from earlier generations in the choice of animal products. However, the same is true in only three out of seven columns in the table on vegetable ingredients (Table 2, fourth table from top), which means that vegetables pose an interesting intermediate case between animal products and spices, perhaps due to the introduction of novel vegetables from the Americas during the span of the investigated period. Also note that meat, fish and fowl are main ingredients in all recipes whereas vegetables and spices are not. Thus *that* animals are used in the cooking we have analyzed is a given, even though *what* animal products that are used varies.

Our results indicate that different ingredients change differently over time; that it is possible to trace different modes of social learning in the chronologically sequenced assemblages. That spices—i.e. tastes—are more prone to be influenced by e.g. fashion and prestige may not be surprising. The conservative character regarding the choice of animal products is in marked contrast with those associated with the vegetables; the transition from the pre-modern, pre-industrial era is most pronounced in this category. The use of vegetable ingredients in fish, red meat and poultry dishes seems to be very varied. It is important to remember that we have analyzed ingredients in fish, red meat, and poultry dishes only and not complete meals. The common, periodically dominating, carbohydrate-rich staple foods and other accompaniments and side dishes are thus not included.

As stated in the results, the rates of change vary in the investigated material. Between the two medieval cookery books, from 1200 and 1390, the general changes are rather small. The largest change per time unit is in vegetables, an effect depending on a great increase in the number of vegetable ingredients. The supposedly great changes of the Renaissance are not reflected in any changes seen between the cookery books of 1390 and 1650. The changes per time unit are in fact the smallest we found in this study, the largest change found in the category of animal products. Investigating the data more closely, there are many small changes in animal products and one striking large change in the rather drastic increase of butter between these two cookery books. Between the cookery books from 1650 and 1739 there are relatively large changes occurring per time unit, the largest of these is to be found in vegetables and the smallest in spices (Table 3).

The most striking change is the increased use of bread and grated bread in the dishes, presumably as thickener. As mentioned above we saw an increase in most of the measures we recorded in the earlier study [15], with increases in the number of steps, techniques, ingredients, and semi manufactured ingredients over time. Though just a trend in the overall picture, the recipes from the 1650 and 1739 cookery books fell below the regression lines in a number of aspects; the number of steps, processes, ingredients and semi manufactured ingredient. The period between these two cookery books is a turbulent one in Europe, spanning the Age of Enlightenment, the Baroque and the era of great food conflicts (ending with the French revolution and/or the start of the Napoleonic Wars). For good or bad, the turmoil of the period certainly increased the interaction between people and the exposure to new social environments, things that may have increased the exchange of ideas also regarding food and eating [32].

Between 1739 and 1879 the largest change is again in vegetables (Table 3). Conspicuous changes in the raw data is a decreased use of sugar and of bread, the later maybe due to the preference of flour for thickening, since flour is increasing. Here, tomatoes show up for the first time in our material. The period between these two cookery books saw both the end of the enlightenment, the French Revolution, the Napoleonic Wars and, more importantly, the introduction of the iron rage and of canning. The decreased use of sugar in fish, red meat and poultry dishes is interesting since sugar was no longer a luxury food. This could possibly be associated with a new thinking in relation to the physiology of nutrition that permeate the 1879 cookery book.

Between 1879 and 1976 the largest change is to be found in spices (Table 3). In the period between, there occurred great changes in cooking and preservation techniques (i.e. refrigeration, electrical stoves, etc.), but also changes in the expression of social difference as well as in foodstuffs. The eye-catching changes in our data are the emergence of white pepper and dill, accompanied by the disappearance of the bouquet and of nutmeg. That social differences came to be expressed more through quality and style rather than quantity and kind fits well with a pronounced change in the use of spices. The long historical domination of cereals is broken and meat consumption is gaining. In our earlier study [15] we found a decrease in the number of self-made semi manufactured ingredients between these two cookery books, breaking with a very old trend of increase. Not producing self-made semi manufactured ingredients on any large scale is a very modern phenomenon. There seems to be no such break in the general use of semi manufactured ingredients indicating that in modern society semi manufactured products are to a greater extent purchased rather than produced at home.

Between the cookery books from 1976 and 1999 we found the largest changes per time unit in the whole data set, again with the largest change found in spices now followed by the animal products (Table 3). The evident number of spices is almost doubled, increasing by ~85%. Among the animal products an increase of chicken is noticeable, as is a drastic increase of fresh milk accompanied by a decrease in the use of cream. The extensive use of fresh milk in cooking is a clearly modern phenomenon. It should be noted that the time distance in time between these two cookery books is the shortest in the whole study. This may have had an effect on the calculation of change per time unit simply because the denominator here is significantly smaller in comparison with those between the other cookery books. Investigating how much of the difference between cookery books that is due to the time factor effect showed that at an estimated natural background variation of 90% of the minimal distance (the least amount of difference between assemblages) between any two books in the matrix these high rate of change is still pronounced except for the vegetables which are still pronounced at 50%. Generally the great increase in change may therefore in fact be real, perhaps an indication of increasing global connectedness. The period saw a dramatic internationalization, commercialization and also normalization of eating. The increase of number of ingredients [15] is probably not surprising with the current global food market and modern preservation and transport possibilities. But measured in number of ingredients per recipe it also indicates more complex mixtures than before. The present study further shows that the increase in complexity observed earlier [15] is very likely accompanied by an increase in the rate of change in the distribution of ingredients in recipes in the analyzed sequence of northwestern European cookery books.

## Supporting Information

S1 Appendix A selection of ingredients extracted from the cookery books of this study. (DOCX)
