# Supplementary material for: A Novel Method to Analyze Social Transmission in Chronologically Sequenced Assemblages, Implemented on Cultural Inheritance of the Art of Cooking
Source: PLoS One. 2015 May 13;10(5):e0122092. doi: 10.1371/journal.pone.0122092 (PMC4430218; doi:10.1371/journal.pone.0122092)
Supplement: S1 Appendix — (DOCX) [file pone.0122092.s001.docx]

# Appendix S1

# Tables

Appendix table 1. A selection of ingredients extracted from the cookery books of this study (Table 1), setting a threshold of occurrence in at least 10 percent of the recipes of any given cookery book. In this selection the three categories of spices, animal- and vegetable products, as specified above, were investigated more closely. Ingredients more commonly present in the early cookery books but decreasing in frequency are found high up in the table while ingredients increasing in popularity with time are found lower down in the table.

| **Spices** | **Animal products** | **Vegetable products** |
| --- | --- | --- |
| sage | pork | wheat bread |
| saffron | hen | garlic |
| cinnamon | eel | pine nuts |
| cumin | pike | raisins |
| ground ginger | yolk | almond |
| powdour fort | egg | bread |
| herbs | capon | sugar |
| ginger | blood | currants |
| parsley roots | salmon | grated bread |
| mace | ox meat | apples |
| nutmeg | lamb meat | shallot |
| clove | chicken | truffels |
| bouquet | goose | rice |
| pepper | milk | tinned mushrooms |
| curry | larding bacon | tinned tomatoes |
| white pepper | broiler | onion |
| white pepper corns | fish | mushrooms |
| dill | minced meat | tomatoes |
| parsley | beef | lemon |
| bay leaf | butter | flour |
|  | cream | carrot |
